# Stencil Lithography for Scalable Micro- and Nanomanufacturing

**DOI:** 10.3390/mi8040131

**Published:** 2017-04-19

**Authors:** Ke Du, Junjun Ding, Yuyang Liu, Ishan Wathuthanthri, Chang-Hwan Choi

**Affiliations:** 1 Department of Mechanical Engineering, Stevens Institute of Technology, Hoboken, NJ 07030, USA; kdu1@berkeley.edu (K.D.); jding1@stevens.edu (J.D.); tcliuyy@gmail.com (Y.L.); Ishan.Wathuthanthri@ngc.com (I.W.); 2 Department of Chemistry, University of California, Berkeley, CA 94720, USA; 3 Northrop Grumman Mission Systems, Advanced Technology Labs, Linthicum, MD 21090, USA

**Keywords:** stencil lithography, scalable, micropatterning, nanopatterning

## Abstract

In this paper, we review the current development of stencil lithography for scalable micro- and nanomanufacturing as a resistless and reusable patterning technique. We first introduce the motivation and advantages of stencil lithography for large-area micro- and nanopatterning. Then we review the progress of using rigid membranes such as SiN*x* and Si as stencil masks as well as stacking layers. We also review the current use of flexible membranes including a compliant SiN*x* membrane with springs, polyimide film, polydimethylsiloxane (PDMS) layer, and photoresist-based membranes as stencil lithography masks to address problems such as blurring and non-planar surface patterning. Moreover, we discuss the dynamic stencil lithography technique, which significantly improves the patterning throughput and speed by moving the stencil over the target substrate during deposition. Lastly, we discuss the future advancement of stencil lithography for a resistless, reusable, scalable, and programmable nanolithography method.

## 1. Introduction

In recent decades, progress in micro-electro-mechanical systems (MEMS) and nano-electro-mechanical systems (NEMS) has significantly impacted the research and development of wireless communication [1,2], inertial sensing [3,4], optics [5,6], biomedical engineering [7,8], and new energy [9,10]. Because the performance of the MEMS and NEMS devices relies heavily on fabrication techniques, there is a strong demand to develop scalable and inexpensive fabrication techniques. The traditional micro- and nanofabrication techniques typically start with spinning photoresist on a flat and rigid substrate, followed by defining structures with photolithography or electron beam lithography (EBL). After patterning photoresist, thin film deposition and etching are used to transfer the pattern and fabricate the device. Traditional micro- and nanofabrication techniques are designed to pattern on rigid substrates, such as silicon wafers. Patterning micro- and nanostructures on unconventional substrates, such as soft and flexible materials (e.g., polydimethylsiloxane (PDMS)) and curved substrates [11,12], emerged in recent years because of applications in wearable electronics [13], implantable medical devices [14], and disposable sensors [15]. Also, as the required resolution reaches down to a nanometer scale, traditional patterning techniques such as EBL are slow and expensive to pattern in a large area. These problems limit the scalability of the nanodevices.

In recent years, non-conventional lithography techniques have been introduced to address those problems, including nanoimprint lithography (NIL) [16,17], nanosphere lithography (NSL) [18,19], X-ray lithography [20,21], laser interference lithography (LIL) [22,23], dip-pen lithography (DPL) [24,25], and stencil lithography [26,27]. NIL is a scalable nanolithography technique, but it still requires the coating of photoresist materials on the target substrate. Furthermore, heat treatment could affect sensitive substrates, and additional processes are required to remove residual photoresist after imprinting. NSL does not require an expensive setup, but the uniformity and dimension are difficult to control. X-ray lithography requires an expensive X-ray light source and needs well-trained personnel for operation. LIL has been widely used to make periodic patterns, but it is difficult to pattern complicated nanostructures. DPL can easily draw complicated patterns, but the scanning speed is relatively slow.

Compared with other emerging nanolithography techniques, stencil lithography is a resistless, scalable, and reusable nanolithography technique that has high throughput. The use of stencils is among one of the oldest technologies in human history, for which evidence is found from over 35,000 years ago [28]. The concept of using stencils is to transfer the features from a thin sheet, such as paper, wood, or polymer, to the underlying substrates. Because the stencil can be reused on different objects, the defined patterns on the substrates will have the same feature size. This concept has been used to develop stencil lithography for micro- and nanopatterning. Stencils made by lithography and etching allow for the transfer of atoms or ions from the aperture to the target substrates, either by physical vapor deposition or plasma etching.

For stencil lithography, there is no need to coat photoresist on the target substrates. Photoresist is commonly used in standard photolithography; however, this procedure uses toxic solvents, such as gamma-butyrolactone and cyclopentanone, and thus requires a ventilation system during the coating process [29]. It also affects biological samples that are sensitive to solvents. Heat, which causes the delamination of photoresist from unconventional substrates such as PDMS due to the large thermal expansion coefficient difference, is also needed for photoresist crosslinking. After UV exposure, un-patterned photoresist needs to be removed by a developer solution that could also dissolve water soluble substrates. Stencil lithography, on the other hand, can be placed over the substrate with a certain space, which does not cause interfacial problems with the substrate.

Micro- and nanostencils can be reused, which decreases the fabrication costs. As stencils are placed over the substrate, they can be removed after patterning and placed on a new substrate. Instead of scanning high-voltage electrons on the photoresist in EBL for every different sample, the stencil can be made to cover the whole substrate and pattern the micro- and nanostructures with one-time deposition [30]. This significantly reduces the complexity and sample preparation time. In addition, stencils can be placed on a certain location on the substrate or moved over the substrate to pattern micro- and nanostructures on a certain location of the substrate.

Based on the materials’ properties, two types of stencil lithography techniques have been introduced. The first type of stencil lithography uses a rigid stencil mask, such as Si and SiN*x*. The other type uses a flexible membrane, such as polyimide film, PDMS membrane, and photoresist layers. Based on the motion of the stencil mask, stencil lithography can be divided into either static or dynamic mode. In this paper, we review the development of rigid and flexible stencil lithography technologies and discuss the recent advances of dynamic stencil lithography. We will also discuss the future of next-generation stencil lithography for a resistless, reusable, scalable, and programmable nanolithography method.

## 2. Rigid Stencil Mask

### 2.1. Silicon Nitride Membrane

In recent years, the development of metamaterials has allowed the detection of low concentration biomarkers based on the spectral shift at Tera-hertz frequencies (THz) [31,32]. There is an increasing interest to pattern such metamaterials on low-cost and disposable substrates for point-of-care (POC) applications. An ideal substrate would be paper, which is lightweight and disposable. However, it is impossible to pattern such microstructures on paper with traditional patterning techniques.

Tao et al. [33] utilized a SiN*x* microstencil to pattern metamaterials on a paper substrate, allowing the paper to sensitively detect glucose and urea. A SiN*x* film (500 nm in thickness) was deposited on a silicon wafer, followed by patterning of the SiN*x* film with traditional surface micromachining techniques. The silicon wafer was etched from the backside with KOH, resulting in a free-standing SiN*x* membrane. The SiN*x* microstencil was placed on top of the paper substrate, followed by the deposition of a thin gold layer to construct metamaterials. SiN*x* is an ideal material for stencil lithography due to the great thermal stability, mechanical strength, and chemical inertness.

**Figure 1:**
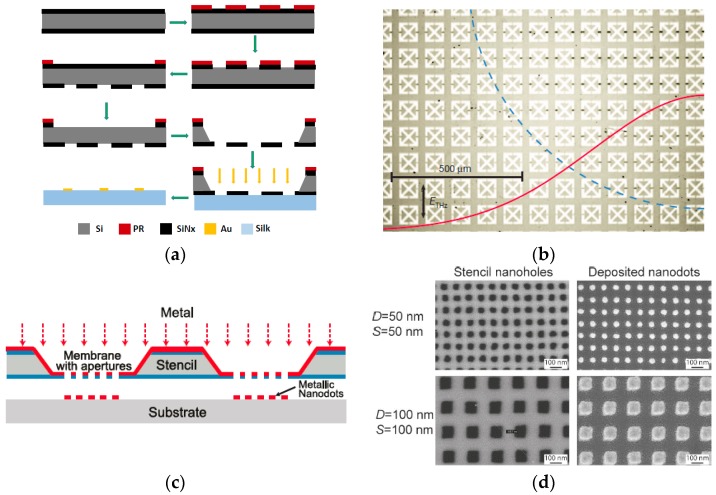
(**a**) Fabrication process of a SiN*x* stencil mask for silk-based sensing platform; (**b**) micrograph of a metamaterials sensor fabricated by using SiN*x*-based stencil lithography. Reprinted by permission from Macmillan Publisher Ltd.: [Nature] [38], copyright (2012); (**c**) schematic image of the nanodots patterning process based on SiN*x* stencil mask with nanoscale features. Reprinted with permission from [39]. Copyright (2010) American Chemical Society; (**d**) scanning electron microscope (SEM) images of stencil nanofeatures and their corresponding metal nanodots with different widths patterned on Si substrates. The deposited nanodots reproduce the shape of the stencil apertures achieving nanodots with a dimension of ~50 nm. Reprinted with permission from [39]. Copyright (2010) American Chemical Society.

Similarly, metamaterials could also be patterned onto silk composites by the stencil lithography technique using a SiN*x* stencil [34,35]. A schematic drawing of patterning metamaterials on silk substrates using SiN*x* stencil lithography is shown in Figure 1a. Silk composite is flexible, biocompatible, transparent, and water-soluble [36,37]. All these advantages are critical for next-generation implantable microdevices. A biosensor patterned on silk substrate can be placed on a curved surface for in situ detection, which is difficult to achieve without using stencil lithography. One of the metamaterials devices patterned by SiN*x*-based stencil lithography is shown in Figure 1b [38].

The resonant frequency of metamaterials can be shifted to visible light by decreasing the feature size of metal structures [40,41]. Such uniform localized surface plasmon resonance (LSPR) sensors can be patterned onto transparent substrates such as glass and PDMS without using photoresist and excessive heat (Figure 1c) [39]. The nanofeatures of the stencil membrane were defined by using EBL and dry etching on a low-stress silicon nitride layer (100 nm) deposited on a silicon substrate and then the silicon substrate was etched from the backside with KOH. Uniform gold nanodots with diameters ranging from 50 to 200 nm were conveniently fabricated by using silicon nitride membrane-based stencil lithography (Figure 1d) [39].

It is possible to pattern metal micro- and nanostructures onto flexible substrates by using other techniques such as pattern transfer [42,43,44]; however, the surface properties have to be characterized. An adhesion layer and heat are normally applied between metal patterns and the target substrate to improve transfer [45]. Such processes are not necessary for stencil lithography patterning, which is critical for patterning on sensitive substrates.

### 2.2. Silicon Membrane

**Figure 2:**
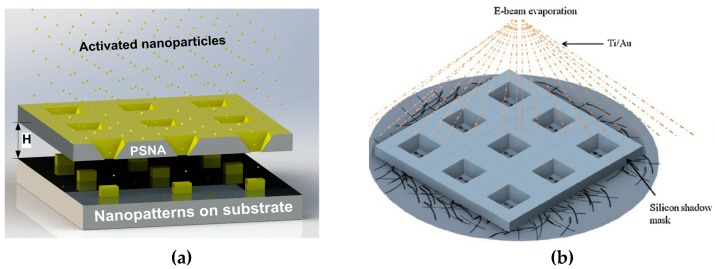
(**a**) Silicon nanostencil mask with pyramidal silicon nanopore arrays (PSNAs). Reprinted with permission from [48]. Copyright (2014), American Chemical Society; (**b**) silicon shadow mask for metallization of single-walled nanotubes (SWNTs). Reprinted from [52], Copyright (2016), with permission from Elsevier.

Low-stress SiN*x* membranes have been widely used as rigid stencil masks; however, silicon membranes are becoming an increasingly popular choice for stencil masks [46,47]. First, an aspect ratio of over 50:1 can be achieved on a silicon membrane by using deep reactive ion etching (DRIE). Due to the great anisotropic etching of silicon materials, a silicon stencil mask can be prepared with a larger thickness than a silicon nitride mask. Thus, a silicon membrane is easier to fabricate and handle. Although the intrinsic stress in silicon membrane is higher than that in silicon nitride, increasing the film thickness could prevent the formation of cracks. Secondly, a single crystalline silicon membrane can be wet-etched by a potassium hydroxide (KOH) solution. Wet etching can be operated in an ambient environment, which significantly reduces the costs of the process. In addition, wet etching on a single crystalline silicon allows for variable aperture diameters and cone angles. Thus, sub-50-nm apertures can be patterned on silicon stencils by using standard photolithography process. Deng et al. [48] fabricated such pyramidal silicon nanopore arrays by using photolithography and wet etching. A 4 µm by 4 µm feature size was first defined by using photolithography, followed by wet etching of silicon via KOH. The chemical wet etching of a P-type (100) silicon wafer resulted in the formation of pyramidal nanopore arrays with a 20-nm pore size. Afterwards, the silicon stencil was used for metal patterning. The schematic of the stencil of pyramidal silicon nanopore arrays is shown in Figure 2a.

The characterization of nanoelectronics such as carbon nanotubes and graphene-based transistors heavily relies on the stability and minimization of contact resistance [49]. Metallic contacts to these nanomaterials can be patterned with stencil lithography without using complicated processes such as ultrasonic welding [50] or molecular linkers [51]. Agarwal et al. [52] used a silicon stencil mask to pattern electrodes on the surface of single-walled carbon nanotubes (SWNTs). SWNTs were first deposited on the target substrate and then a silicon stencil mask was placed on top of the SWNTs. Electrodes were deposited through the stencil on the surface of the SWNTs. After patterning electrodes with stencil lithography, the electrical properties of the SWNTs could be characterized by measuring the resistance of the patterned electrodes. The patterning process just requires one step without additional coating and patterning of photoresist on the SWNTs. A schematic of the electrode patterning on SWNTs is shown in Figure 2b.

### 2.3. Rigid Stencil Stacking

**Figure 3:**
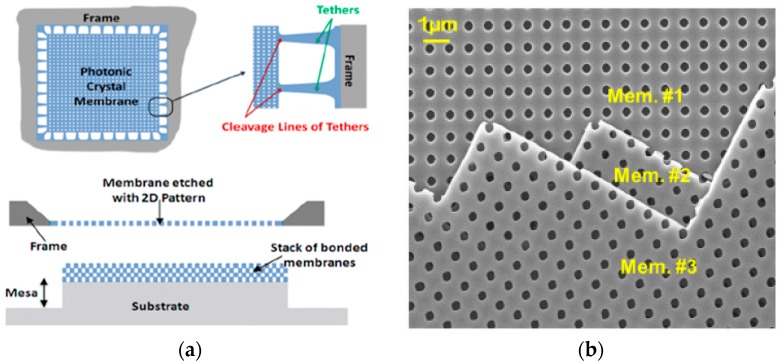
(**a**) Schematic of the membrane stacking approach using the cleavage of tethers from the frame. Reprinted with permission from [55]. Copyright [2011], American Vacuum Society; (**b**) SEM image of a stack of three silicon membranes. Reprinted with permission from [57]. Copyright [2011], American Vacuum Society.

As the patterned stencil mask is relatively thin and covers a large surface area, it can be used as a building block for 3D photonic crystal structures, in addition to the mask for deposition or etching processes. Photonic crystals have been widely studied for their applications in manipulating light, such as optical waveguides and filters [53,54]. The traditional method of fabricating 3D photonic crystal relies on the slow and expensive layer-by-layer process. Instead, Patel et al. [55,56] used SiN*x* membranes as building blocks and stacked the free-standing membranes on the substrate as 3D photonic crystals. The nanoscale features on the SiN*x* membrane were defined by interference lithography followed by dry etching. The SiN*x* membrane was connected with an outer frame by tethers (Figure 3a) [55]. When the membrane is pressed on top of the target substrate, mechanical forces were used to break the tethers and release the membranes. By using the tether design, it is easy to stack several SiN*x* membranes to form a 3D photonic crystal.

Another way to release and stack the stencil membrane is to dissolve the underlying sacrificial layer in the liquid and float the membrane [57]. In this case, because the membrane exhibits little intrinsic stress, it can bond and stack easier. Ghadarghadr et al. [57] showed that 3D nanostructures could be made by stacking a single crystal silicon membrane. After patterning the single crystal silicon layer, the underlying silicon oxide layer was etched by using hydrofluoric acid (HF). This results in the floating of patterned silicon membrane on the free surface of the solution, which can be stacked for further device fabrication. A scanning electron microscope (SEM) image of the stacked silicon membranes is shown in Figure 3b [57]. This powerful technique has been used to achieve a single-mode waveguide over a wide spectrum [58,59].

## 3. Closing and Blurring Effects

**Figure 4:**
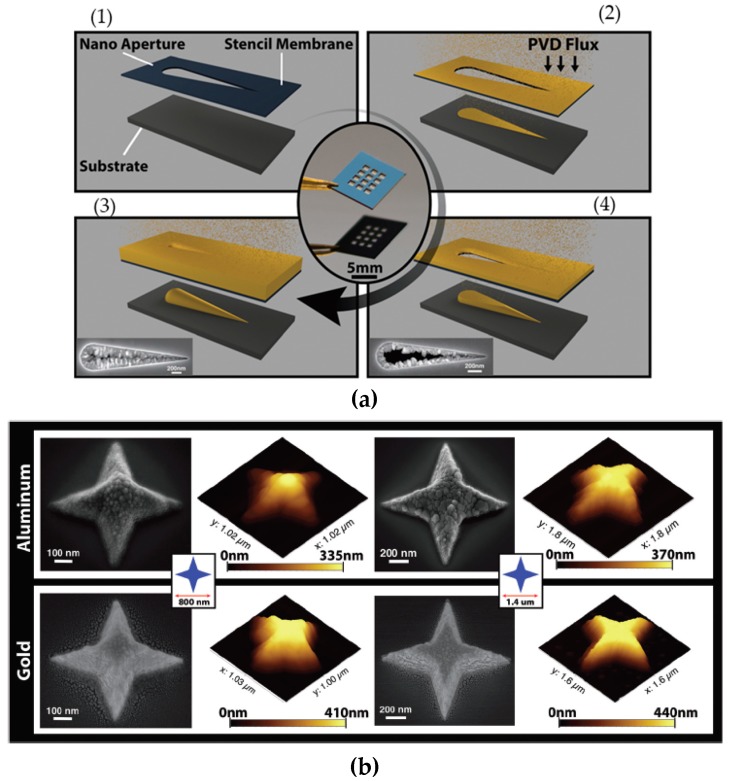
(**a**) Closing effect in stencil lithography: (1) assembly of the stencil mask on a substrate; (2) metal deposition through the open aperture; (3) partial clogging of the stencil mask; (4) full closure of the stencil mask. Reproduced from [64] with permission of The Royal Society of Chemistry. (**b**) Four-pointed star-shape nanostructures with 3D topography. Top and bottom rows represent both the SEM images and the atomic-force microscopy (AFM) topography of the Al and Au nanostructures, respectively. The nanostructure is shown in two different scales: the smaller pattern (left) with 800 nm and the bigger pattern (right) with 1.4 μm in total width. Reproduced from [64] with permission of The Royal Society of Chemistry.

Although stencil lithography has several advantages over conventional lithography techniques, there are a few disadvantages. One of the common problems in stencil lithography is a closing effect [60,61]. The closing effect occurs during deposition. As atoms are deposited through stencil masks, the path should be perpendicular with the target substrate. However, the atoms can spread from the deposition source with a certain angle. This causes the clogging of the deposition materials around the aperture of the stencil, as shown in Figure 4a. After patterning the substrate with the same stencil for several times, more materials are deposited on the aperture, which reduces the feature size to be transferred to a target substrate. In order to resolve the closing problem, several strategies have been developed. A thin coating of a self-assembled monolayer (SAM) reduces the adhesion of depositing materials around the aperture and improves the throughput by as much as 240% [62]. After deposition, it has been found that the deposited materials were built on top of the stencil rather than clogging inside the aperture, increasing the life-time of the stencils [62]. Other techniques such as wet etching can be used to remove the clogging materials, such as gold and aluminum, for later applications [63].

Even though clogging could be a problem for stencil lithography, it could also bring unique opportunities for nanofabrication. For example, as the aperture size of the stencil mask decreases because of clogging, it is possible to fabricate nanostructures with smaller features than the original lithography design. In addition, during the deposition, the feature size becomes smaller. This results in the thicker deposition in the lower part of the nanostructures and thinner deposition in the upper part. This closing effect can be used to make high aspect ratio 3D nanostructures with variable dimensions along the deposition direction. An example of such 3D nanostructures fabricated by using the closing effect is shown in Figure 4b [64]. A four-pointed star-shape is presented in two different dimensions (with total widths 0.8 μm and 1.4 μm, respectively) and materials (aluminum and gold, respectively). The 3D nanostructures profile can be controlled by tuning the parameters in physical vapor deposition.

**Figure 5:**
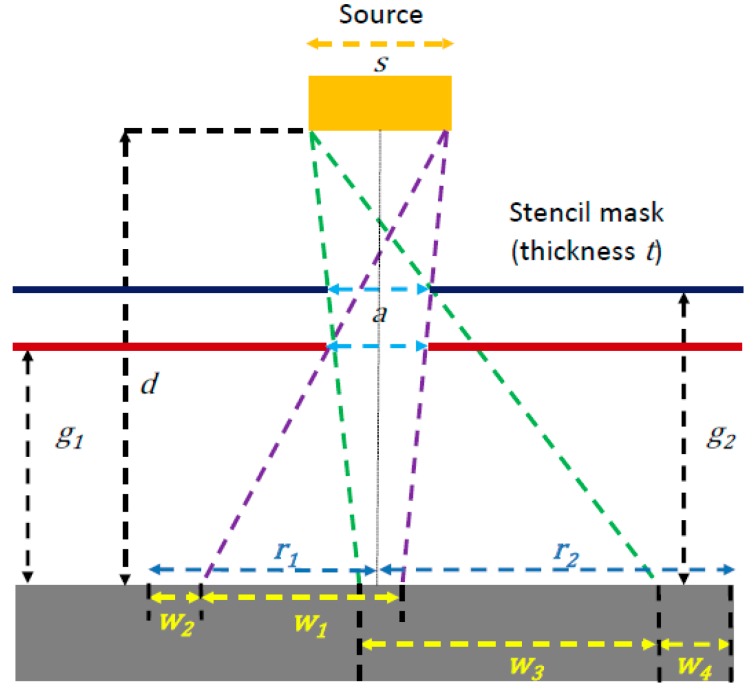
Schematic image of the “blurring” effect. The gap *g* between the stencil mask and substrate increases the feature size based on the shadowing effect.

In a typical stencil lithography patterning, a gap between the mask and the substrate usually exists. The gap reduces the sticking problem of the stencil and the substrate. It also helps to manipulate and align the membrane over the substrate. This is especially important for dynamic stencil lithography, which will be discussed in detail in Section 5, as the contact of the stencil and the substrate could prevent the move of the stencils during deposition. Because of the space between the stencil and the substrate (typically between 10 and 100 µm), the atoms are spread from the aperture to the substrate, which results in a larger feature size compared with the aperture, which is referred to as the blurring effect. A schematic of the blurring effect is shown in Figure 5. The deposition source has a distance, *d*, from the target substrate. The stencil is placed between the source and the substrate. An aperture size of *a* is designed and patterned on the stencil mask. Because of the distance *g*_1_ between the stencil and the substrate, a shadow effect is presented (purple dashed lines); this results in an enlarged feature size of *w*_1_. In addition, a halo (*w*_2_) around the central deposited structure is formed presumably due to the material surface diffusion [65]. The halo is usually thin; therefore, it is hard to notice directly by characterization tools such as SEM or atomic-force microscopy (AFM). The size of the halo increases as the gap between the stencil and the substrate, the thickness of deposition, and the rate of deposition increase [66]. The radius *r*_1_ of the feature size due to the blurring effect is:

(1) $$r_{1}=w_{1}+w_{2}+\frac{\left(s-a\right)d}{2\left(d-g_{1}\right)}-\frac{s}{2},$$

where *w*_1_ represents the enlargement caused by shadow effect, *w*_2_ represents the halo effect, *s* represents the width of the source, *d* represents the distance between source and substrate, *a* represents the feature size of the aperture, and *g*_1_ represents the distance between the stencil and the substrate. The thickness of the stencil, *t*, is assumed to be small compared with the gap size and is neglected.

A larger gap, *g*_2_ (green dashed lines), will cause more significant blurring and enlargement of the structures as shown in Figure 4b. In this case, the radius *r*_2_ is:

(2) $$r_{2}=w_{3}+w_{4}+\frac{\left(s-a\right)d}{2\left(d-g_{2}\right)}-\frac{s}{2},$$

where *w*_3_ represents the enlargement caused by shadow effect, *w*_4_ represents the halo effect, and *g*_2_ represents the distance between the stencil and the substrate with a larger gap. It should be noted that both *w*_3_ and *w*_4_ increase due to the increase of the gap size (*g*_2_).

One of the solutions to reduce the blurring effect is to use a compliant membrane that covers the substrate seamlessly. In this case, the distance *g* disappears, which eliminates the blurring effect.

## 4. Compliant Stencil Mask

As the blurring effect leads to the enlargement of the nanofeatures, developing a stencil lithography technique that can avoid the gap between the stencil and the target substrate during patterning has drawn great attention. By avoiding the gap, it can also reduce the patterning inaccuracy when the target substrate is uneven or curved. The development of a compliant stencil mask helps to reduce the blurring effect between the stencil mask and the substrate by putting the stencil mask in contact with the target substrate. Due to the high flexibility and ductility of the flexible membranes, it also reduces the risks of fracture or damage during patterning. Here, we summarize the recent advances of compliant stencil lithography techniques.

### 4.1. Rigid Membrane with Compliant Cantilevers

**Figure 6:**
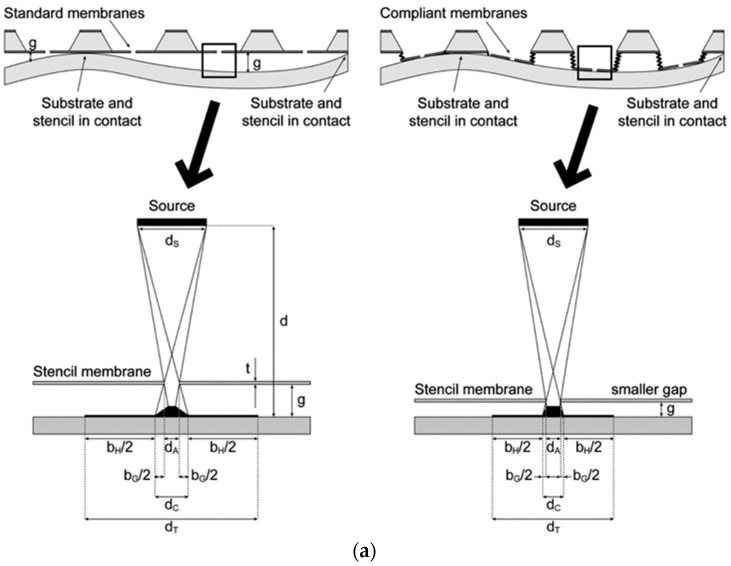

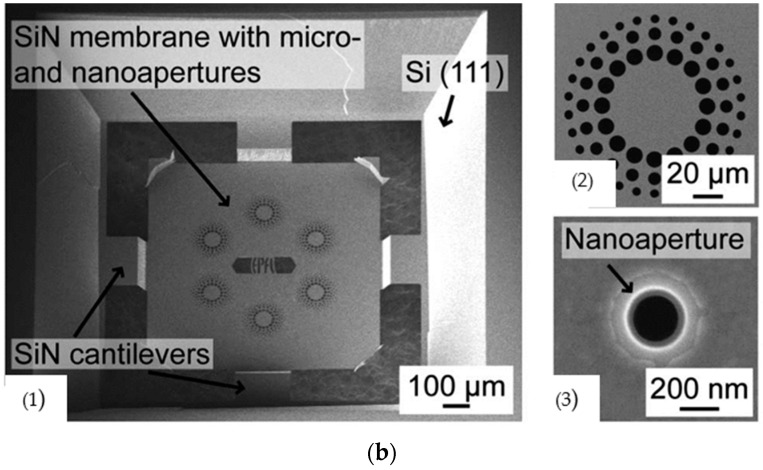
(**a**) Feature size of d_Ʈ_ with a gap distance of *g* (left). Feature size of d_Ʈ_ with a gap distance of *g* by using a compliant stencil mask. Reproduced from [68] with permission of The Royal Society of Chemistry. (**b**) SEM image of a free-standing compliant membrane suspended by four cantilevers (1) and the images of nanoapertures on the SiN*x* membrane (2,3). Reproduced from [68] with permission of The Royal Society of Chemistry.

Cantilever structures can be made by using standard micromachining techniques [8]. The mechanical properties of the cantilevers, such as flexibility, can be controlled by adjusting the geometry and material properties of the cantilevers. The deformation of the cantilevers due to a mechanical load can be determined by experiments or finite element modeling (FEM). Using cantilevers, the rigid stencil mask can be pressed into full contact with a substrate by elastically bending the cantilevers. As the cantilevers are flexible, the rigid membrane can adapt to the surface irregularity and reduce the blurring effect. Sidler et al. [67] used the flexible and protruding SiN*x* cantilevers connected to a rigid membrane as a compliant stencil lithography mask. Four 200 µm long SiN*x* (100 nm in thickness) cantilevers were fabricated, which could deflect 40 µm under a load of 45 µN [67]. In Figure 6a, the comparison of a traditional stencil lithography (left) and the cantilever assisted stencil lithography (right) is shown [68]. Due to a smaller gap *g* in the compliant cantilever mode, the blurring effect is minimized. Comparing with the rigid stencil mask, 40% blurring reduction was observed for the compliant stencil, with an aperture size ranging from 200 to 1000 nm. The SEM image of the compliant cantilever-based stencil lithography is shown in Figure 6b1 [68]. High-magnification SEM images of the nanoapertures are also shown in Figure 6b2,b3 [68]. The cantilever-assisted compliant stencil lithography significantly reduces the blurring effect by reducing the gap size *g*; however, the addition of flexible cantilevers increases the complication of the fabrication processes, which will require careful characterization of the deformation of the cantilever beams. Therefore, a stencil mask made of flexible materials is in high demand.

### 4.2. Polyimide Film

**Figure 7:**
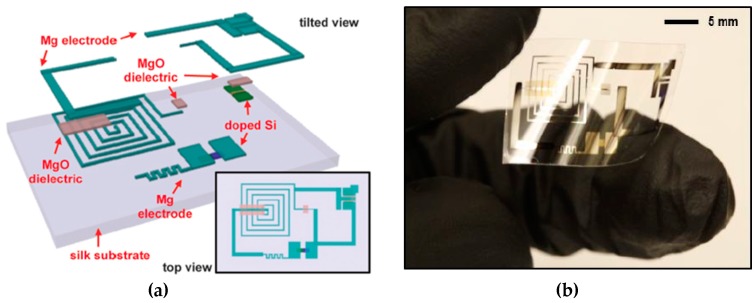
(**a**) Schematic of fabrication of process of transient electronics by using stencil lithography. Mg and MgO were patterned on silk substrate sequentially with stencil lithography. From [73]. Reprinted with permission from AAAS. (**b**) Image of a flexible transient electronic device patterned by using polyimide based stencil lithography. From [73]. Reprinted with permission from AAAS.

Polyimide films are flexible and resistant to heat and chemicals. They have been used as the substrate for printed electronics and organic thin film transistors [69,70]. Since polyimide films are very flexible, they could be attached to the patterning surface and serve as a peel-off stencil mask. In recent years, biodegradable electronics have drawn great interest due to their applications in implantable medical devices and environmentally friendly sensors [71,72]. In making such electronic devices, the circuits must be patterned onto water-dissolvable substrates, such as silk composites. Because excessive heat and a chemical etchant are needed for conventional electronics patterning, sensitive substrates could be damaged during device fabrication. Hwang et al. [73] used a polyimide film as a stencil mask to pattern such physically transient electronics that can be destroyed by the environment (e.g., water) over a certain time of period. In their work, a polyimide film with a thickness of 12.5 µm was attached to a PDMS coated glass slide. Then, a thin metal layer was deposited on top of the polyimide film and patterned with photolithography. The metal film was wet-etched and served as an etching mask for the polyimide film. The polyimide film was etched by oxygen plasma and peeled off from the glass slide as a stencil mask. They used a high-resolution stencil mask to pattern dielectric materials (MgO and SiO_2_), as well as a metal electrode on top of the biodegradable electronics. The schematic drawing of the device layout and image are shown in Figure 7 [73]. This shows that, without necessitating the coating and lift-off processes of photoresist for every layer of a device, the flexible stencil lithography mask can be attached to the device and used to deposit materials. The whole fabrication process is simpler, especially for multi-layer structure patterning.

Although a polyimide film can be used as a stencil mask for patterning, the thickness of the commercially available films is between 7 and 125 µm. Patterning nanoscale features by using the commercially available polyimide films is difficult; etching through a thick polyimide film with nanoscale aperture is challenging because of the isotropic etching property. As the aspect ratio (film thickness: feature size) increases, it becomes more difficult to make such stencils. Therefore, there is a strong need for flexible and nanoscale-thickness stencil masks.

### 4.3. PDMS Membrane

PDMS materials have been widely used in flexible electronics [74], microfluidics [75], and optical waveguides [76]. The silicone-based organic polymer is soft, bendable, transparent, and has great chemical inertness. All these advantages have made PDMS a strong candidate as a stencil mask.

Meng et al. [77] used PDMS stencil masks to pattern on water-soluble cellulose nanofibril paper substrates. Such paper is made from a wood-derived cellulose nanocrystal (CNC) and is further used as a transient electronics substrate. In their work, PDMS was poured onto an etched silicon wafer. Subsequently, a doctor blade was used to remove excess PDMS from the top silicon surface to make an open trench. After curing, the PDMS membrane was peeled off from the silicon wafer, pressed on top of the CNC paper, and used as a stencil for electrode patterning. By using this technique, they were able to make a strain sensor with a resistance that is linearly related to the elongation of the CNC paper. Since PDMS is ductile and does not produce cracks during handling, it could be used for numerous cycles. In addition, since PDMS is chemically inert, it is an ideal material to be used as a stencil mask for etching [77].

**Figure 8:**
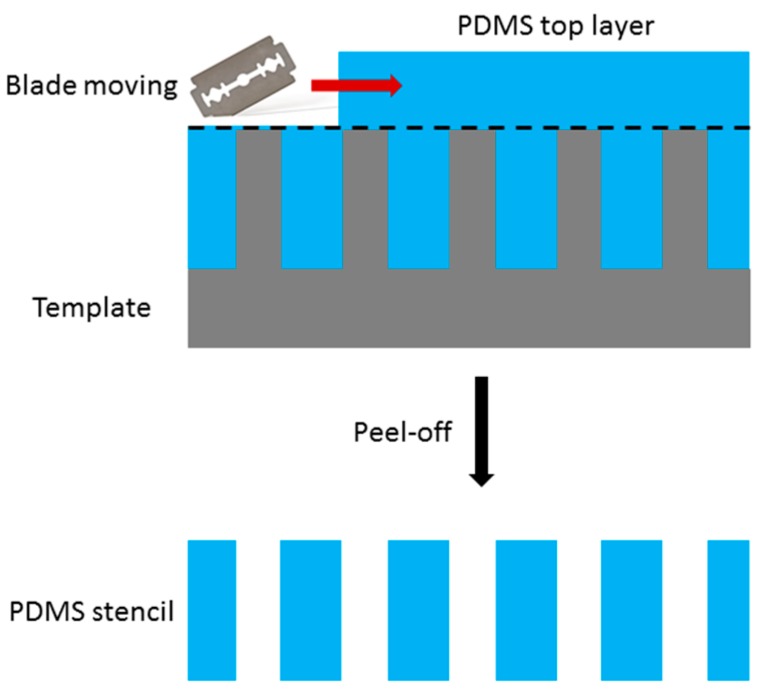
Schematic of fabrication of polydimethylsiloxane (PDMS) stencil by using a doctoral blade.

However, creating an aperture in PDMS is very challenging. Since PDMS is poured onto the microstructures, the top layer needs to be removed using a doctor blade [77]. A scheme of the creation of a PDMS stencil is presented in Figure 8. After the curing of PDMS, a sharp blade has to be used to remove the top layer of PDMS to open the aperture. This could lead to damage to the mold and it is difficult to locate the cross section for the removal. After removal of the top layer, the stencil can be peeled off from the template. As removing the top layer is typically done manually, removing the top surface of nanostructures is extremely difficult. Other techniques such as applying negative or positive pressure on the PDMS film for stencil mask fabrication have been introduced to remove the top layer, but all increase the complexity [78,79]. Since PDMS is inert and it is difficult to etch an aperture through a PDMS membrane, PDMS stencils are not an ideal candidate for nanoscale stencil lithography.

### 4.4. Photoresist Membrane

**Figure 9:**
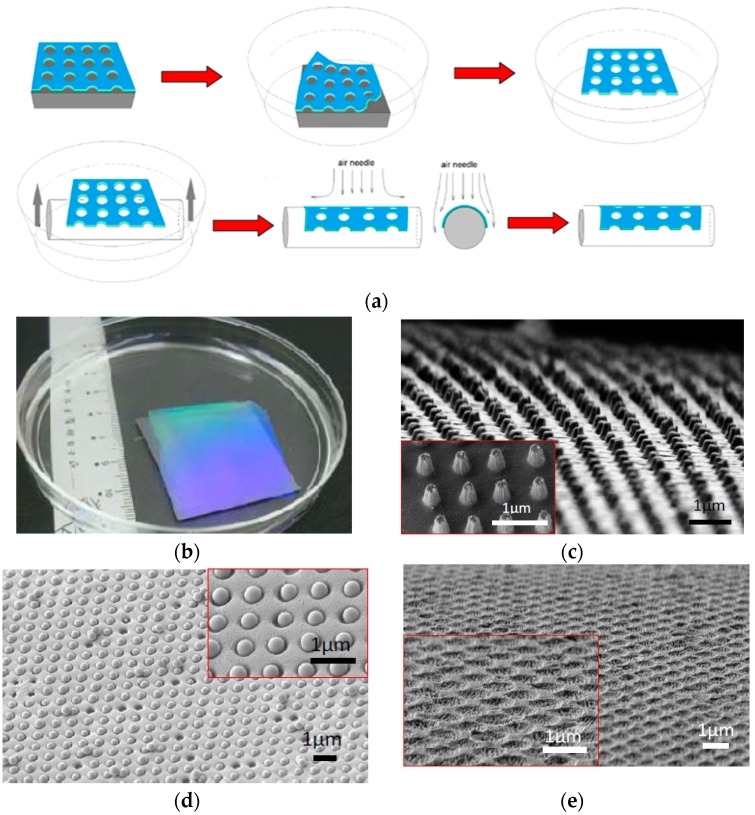
(**a**) Schematic of release and transfer processes of a free-standing photoresist stencil mask to curved substrates. © [2012] IEEE. Reprinted, with permission, from [88]; (**b**) image of a photoresist membrane released and floating in delaminating (bubbling) solution. © [2012] IEEE. Reprinted, with permission, from [88]; (**c**) gold nanopillars featured on a curved surface of an optical fiber using the transfer processes. © [2012] IEEE. Reprinted, with permission, from [88]; (**d**) silicon oxide nanoparticles assembled on the curved surface of an optical fiber through the nanoporous photoresist stencil mask. © [2012] IEEE. Reprinted, with permission, from [88]; (**e**) periodic nanoholes etched on a Poly(methyl methacrylate) (PMMA) optical fiber by using the photoresist stencil mask as an etch mask. © [2012] IEEE. Reprinted, with permission, from [88].

Due to the ineffectiveness of using PDMS as a stencil mask for nanoscale patterning, we have developed a stencil lithography technique for scalable micro- and nanomanufacturing based on free-standing photoresist membranes. Both bi-layer membrane (consisting of antireflective layer and photoresist) and tri-layer membrane (consisting of antireflective layer, photoresist, and metal) were developed and investigated. The nanofeatures on the membranes were created by laser interference lithography, which was able to produce uniform nanopatterns on a wafer scale [80,81,82,83,84,85,86]. The periodicity of the nanostructures can be regulated by the wavelength of the laser and the angle between the two interfering laser beams. The patterning coverage area can also be expanded by longer expansion of the beams with a higher laser power. Recently, Bläsi et al. [87] demonstrated the fabrication of nanostructures of 200 nm in periodicity uniformly over a surface area of 1.2 m × 1.2 m. After the creation of the nanopatterns, a mixture solution of hydrogen peroxide and ammonia were used to release the membrane from the silicon substrate via bubbling [88]. This results in the floatation of the membrane on the solution-free surface (Figure 9a). As the membrane is floating, there is no intrinsic stress, minimizing wrinkles or cracks. Similar techniques using air bubbles have also been demonstrated to release ultrathin 2D materials from the original substrate and transfer to arbitrary surfaces with little damage on the membrane [89,90]. Such flexible membranes have been used for large-area patterning on curved substrates, flexible substrates, and hierarchical micro- and nanostructure patterning.

#### 4.4.1. Curved Substrate Patterning

In recent years, patterning on curved substrates has drawn great attention due to the applications in optofluidics [91], microfluidics [92], and plasmonic sensing [93]. In traditional lithography, a photoresist layer is normally spin-coated on a planar and rigid substrate and used as a planar template for pattern transfer. It is very difficult to pattern periodic micro/nanostructures on a curved surface by using the planar patterning. Although non-planar surface patterning was demonstrated with thermal evaporation of resist followed by electron-beam lithography, such a serial process is slow and expensive for scalable nanopatterning [94,95]. Due to the high flexibility and throughput, the soft nanostencil lithography is an ideal technique for non-planar surface patterning. We have shown that such 3D patterning can be achieved by using bi-layer stencil masks composed of antireflective coating layer and photoresist. The key is to apply the free-standing bi-layer membrane along the curved substrate and use the membrane as a template for physical vapor deposition, self-assembly, or dry etching for pattern transfer. As shown in Figure 9a, an anti-reflective coating (ARC) was spin-coated on a silicon wafer, followed by the spin coating of photoresist layer with a thickness ranging from 300 nm to 1.5 µm [88]. Nanopatterns were defined by using laser interference lithography, which covered the whole wafer area. After that, oxygen plasma etching was used to etch the ARC layer. The membrane was released by a mixture of H_2_O_2_ and NH_3_. The concentration of the mixture was optimized to result in the release of the membrane by bubbling effects [88,90] within one hour. After releasing the membrane in the solution, it was attached to a rod or curved surface by putting the rod under the suspended film and slowly lifting the rod. Pressurized air was used to improve the conformal spreading of the film over the curved substrate. Degassing was then used to remove any possible gap between the membrane and the curved substrate. A micrograph of the free-standing membrane released in the bubbling solution is shown in Figure 9b [88]. This optical fiber with high aspect ratio gold nanostructures was realized by attaching the bi-layer membrane around the rounded fiber surface (Figure 9c). The nanoporous bi-layer membrane covering the curved surface of the optical fiber has also been employed as the template for nanoparticles assembly (Figure 9d), targeting for plasmonic sensing and waveguides. The nanoporous bi-layer membrane has also been utilized as an etching mask to transfer the periodic nanopatterns directly onto the curved surface of the optical fiber (Figure 9e) [88].

#### 4.4.2. Dual Applications of Tri-Layer Membrane

Lift-off is one of the most commonly used techniques for the patterning of metal or oxide layers on rigid substrates [96,97]. In a typical lift-off process, the photoresist is coated and patterned by using lithography. Then, the photoresist is removed and abandoned. Thus the traditional lift-off technique is not ideal for repetitive and scalable production. Stencil lithography, on the other hand, can be used to pattern metal structures on the substrate repeatedly. Recently, we have also developed a process to achieve a tri-layer stencil mask comprised of ARC/photoresist/metal and investigated the use of a tri-layer stencil mask for dual applications to create both negative and positive patterns of the stencil mask via lift-off and dry-etching methods, respectively [98].

**Figure 10:**
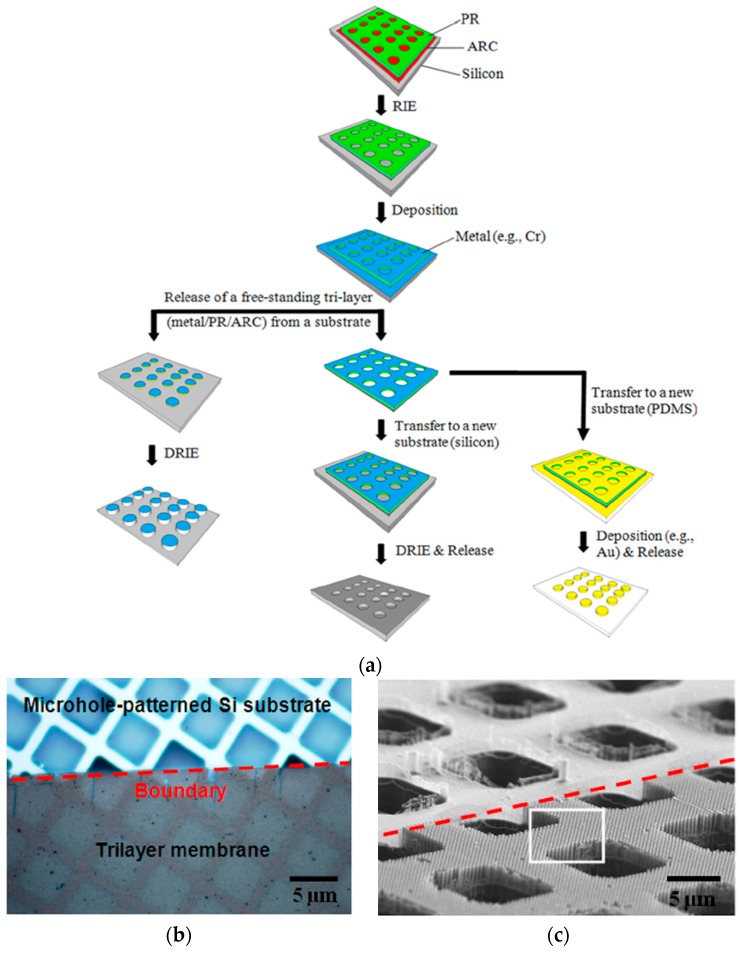

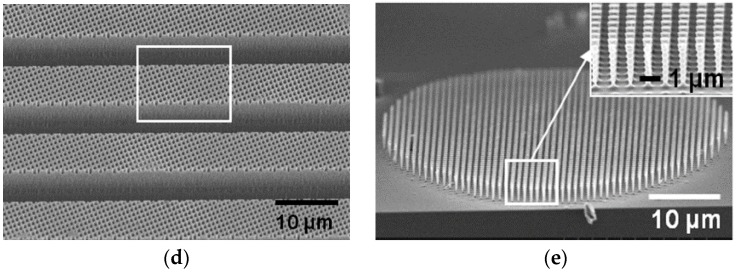
(**a**) Schematic of dual patterning of both nanopillars and nanoholes on silicon substrates by using tri-layer membrane based stencil lithography technique. Reprinted with permission from [98]. Copyright [2012], American Vacuum Society; (**b**) image of a tri-layer membrane placed on microhole-patterned silicon substrate. Reprinted with permission from [110]. Copyright [2013], American Vacuum Society; (**c**) SEM image of the hierarchical nanopillar structures formed on top of the microhole structures. Reprinted with permission from [110]. Copyright [2013], American Vacuum Society; (**d**) SEM image of hierarchical nanohole structures patterned on micrograting structures by using tri-layer membrane. Reprinted with permission from [110]. Copyright [2013], American Vacuum Society; (**e**) SEM image of a micropatch of silicon nanopillars patterned by using SU-8 based micro-membrane. Reprinted with permission from [110]. Copyright [2013], American Vacuum Society.

As shown in Figure 10a, ARC and photoresist were patterned using laser interference lithography and plasma etching, then a thin Cr layer (20 nm) was coated on the silicon surface [98]. Instead of removing the membrane with acetone or another solvent, the ARC/photoresist/Cr tri-layer was released by using the mixture of H_2_O_2_ and NH_3_. The original silicon substrate was patterned with Cr nanodots, which were used as an etching mask. This resulted in high-aspect ratio silicon nanopillars. On the other hand, the released tri-layer membrane was transferred onto a new silicon substrate and used as an etching mask. Deep reactive ion etching (DRIE) was used to etch silicon nanoholes. Therefore, by using the same tri-layer stencil mask, both silicon nanopillars and silicon nanoholes can be patterned. The tri-layer membrane can be released again after the patterning and reused several times. This significantly improves the patterning throughput. Since DRIE process has a high etching selectivity of silicon over Cr, the tri-layer membrane is preserved after etching and can be reused again. Alternatively, the tri-layer can also be put on a flexible substrate such as PDMS and Polyethylene terephthalate (PET) and used as a deposition mask for high-aspect ratio metal nanostructures. The tri-layer membrane is more rigid (mainly due to the top metal layer) than a bi-layer membrane. Consequently, it is less susceptible to the issue of wrinkling during the membrane placement. However, the tri-layer membrane is more brittle than the bi-layer membrane due to the interfacial stress between the metal (Cr) and photoresist. The same tri-layer membrane can be reused 3–4 times as deposition and etching mask with high repeatability and low degradation. However, after a few uses, the intrinsic stresses induced from the deposition or etching creates cracks in the nanostencil, leading to failure. It is regarded that the use of more ductile metal for the top layer, such as gold or silver, would mitigate such issues. Since the tri-layer membrane is coated seamlessly on the target substrate, the blurring effect is significantly reduced. This feature for tri-layer membrane is extremely important when used as an etching mask at nanoscale level because a small gap between the stencil and substrate will cause isotropic etching of the substrate.

#### 4.4.3. Hierarchical Nanostructures Patterning

Hierarchical nanostructures have drawn great attention due to their applications including anti-wetting surfaces [99,100], antireflective surfaces [101,102], and self-assembly of nanomaterials [103,104]. With the addition of high aspect ratio nanostructures, the surface properties of micro- and nanodevices can be improved. The fabrication of well-ordered hierarchical nanostructures is challenging. Dual-scale electron-beam lithography [105], nanomolding [106], and nanoimprint lithography [107] have been applied to make such hierarchical nanostructures. However, the fabrication processes are always time-consuming and expensive. One-step plasma etching can be used to fabricate hierarchical nanostructures; however, the feature size and period cannot be controlled precisely [108,109]. Using the tri-layer membrane as a stencil mask, we have also developed a new fabrication scheme of making large-area hierarchical nanostructures [110].

For example, a nanoporous tri-layer membrane made via laser interference lithography was placed on pre-patterned silicon microstructures (Figure 10b). To create high aspect ratio nanopillars on top of the silicon microstructures, a thin layer of Cr was deposited through the nanopores of the flexible tri-layer membrane. After the release of the membrane, silicon microstructures were etched by using DRIE to form high aspect ratio nanostructures on the top surfaces. In order to create high aspect ratio nanoholes on the silicon microstructures, DRIE was directly applied through the nanopores of the tri-layer stencil mask. The SEM images of the high aspect ratio nanopillars featured on microtrenches and nanoholes on microgratings are shown in Figure 10c,d, respectively [110].

The introduced photoresist membrane as a stencil mask is a multiscale technique. For example, we were also able to release thick (50 µm) SU8/ARC/Cr tri-layer membrane with micropores by using the introduced releasing protocol. For the micropatterned (feature size greater than 2 µm) photoresist membrane, a conventional photolithography was employed. The SU8 based tri-layer membrane with micropores was placed on top of smaller-scale nanostructures and served as a microscale stencil mask for etching. After coating a thin Cr layer and releasing the membrane, we were able to etch micropatches of high aspect ratio nanostructures by using a Cr mask in DRIE etching. Such hierarchical nanostructures are very useful in biomedical applications such as nucleic acid detection, tracking, and patterning [111,112,113]. A SEM image of such hierarchical nanostructures is shown in Figure 10e [110].

## 5. Dynamic Stencil Lithography

**Figure 11:**
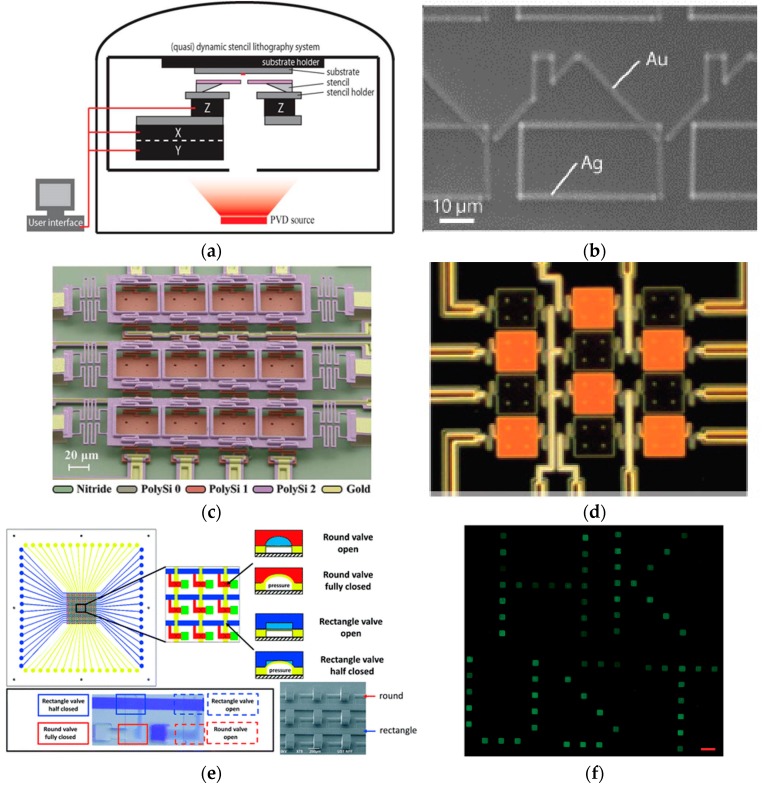
(**a**) Schematic of a dynamic stencil lithography setup. The stencil mask is mounted on a high-precision *XY* stage, while the substrate holder is fixed at a controlled distance from the stencil by Z actuators. Reprinted with permission from [114]. Copyright [2008], American Vacuum Society; (**b**) parallel fabrication of a multiscale house structure by using dynamic stencil lithography. Reprinted with permission from [114]. Copyright [2008], American Vacuum Society; (**c**) a stencil lithography system with integrated deposition sources. Reproduced from [117] with permission of The Royal Society of Chemistry; (**d**) independent actuation of deposition sources for precise patterning of metal atoms. Reproduced from [117] with permission of The Royal Society of Chemistry; (**e**) a digital microfluidic programmable stencil lithography technique for protein and cell patterning. Reproduced from [121] with permission of The Royal Society of Chemistry; (**f**) protein patterning made by using microfluidic programmable stencil lithography. Reproduced from [121] with permission of The Royal Society of Chemistry.

Stencil lithography can also be realized in a dynamic mode. For static stencil lithography, deposition and etching are carried out through an aperture in the stencil mask that is not moving over the substrate. As the position and displacement of the membrane can be precisely controlled with programmable motors and a high-precision XY stage, the stencil mask can move over the substrate during patterning (Figure 11a) [114]. There are several advantages of dynamic stencil lithography. First, dynamic stencil lithography can pattern complicated nanostructures by moving the stencil over the substrate [115,116]. Thus, the patterning shape can be adjusted by programming the moving path of the stencils. Complicated nanostructures that are difficult to pattern using other nanolithography techniques can be achieved. For example, the metal structures (gold and silver) of “rooftop” geometry could be fabricated via dynamic stencil lithography by moving the programmed stencils (Figure 11b) [114]. Secondly, the thickness of the nanostructures can be easily adjusted by modifying the deposition time. By changing the deposition time and moving the stencil, 3D nanostructures with various heights can be easily fabricated, which is important for nanoelectronics and nanooptics applications.

With the development of MEMS technology, it is possible to integrate deposition sources and stencil masks into a single device. A matrix of deposition crucibles can be operated by heating the crucibles independently. The heated sources can evaporate through a nanostencil over the crucible and deposited on the target substrate. Han et al. [117] demonstrated controllable metal deposition ranging over eight orders of magnitude, and successfully deposited different metals such as silver, gold, copper, and aluminum by using a single device (Figure 11c). It can also be operated by a moving stage to achieve dynamic stencil lithography. Figure 11d shows the images of heated element (bright) and un-heated elements (dark), respectively, in using such an approach [117].

Dynamic stencil lithography can also be integrated with digital microfluidic systems for biological samples patterning. In recent years, pneumatically controlled microvalves have been widely used in automated microfluidic systems with the capability of manipulating the low volume of reagents [118,119,120]. The actuated microvalves allow the transfer of fluids from one reservoir to another. By opening an aperture on the bottom substrate, it allows the deposition of regents on the bottom layer. By adding a stencil mask between the microfluidic system and the bottom substrate, it can easily regulate the shape and size of the biological samples on the deposited substrate. Gao et al. [121] developed such microfluidic-based stencil lithography. The design is shown in Figure 11e. The regents were introduced from fluid inlets and the programmable pneumatic microvalves open/close sequentially to allow precise deposition of biological samples. A protein pattern fabricated by digital microfluidic programmable stencil is shown in Figure 11f [121].

## 6. Conclusions

The recent development of stencil lithography has created many advantages in the fields of scalable micro- and nanomanufacturing. Stencil lithography is resistless, thus it avoids the heating and chemical treatment for the photoresist materials’ crosslinking. This allows for patterning on sensitive substrates such as silk and paper. Stencil lithography is scalable with the capability of patterning in wafer scale or even larger area. Stencil lithography is a reusable technique, which significantly reduces the fabrication costs and time. Stencil lithography has now been widely used in the fields of biofabrication [78], biosensing [122], waveguides [58], photonics [41], and flexible electronics [35]. It has also been used to fabricate large-area and uniform master molds for nanoimprint lithography (NIL) and significantly improves the throughput of NIL [123]. Unlike other forms, stencil lithography can move the stencil during patterning. This allows the precise fabrication of complicated micro- and nanostructures by programming the moving path of stencils. However, the presence of the blurring effect affects the patterning resolution. To address this problem, compliant membranes have been introduced, which decreases the blurring effect by seamlessly covering the substrate with a flexible stencil.

Although stencil lithography has demonstrated great potential as a next-generation micro- and nanolithography technique, there are still several challenges. For example, although compliant membranes can reduce the blurring effect, the stencil mask is in close contact with the substrate, which cannot be moved during deposition. Thus it affects the patterning speed. In addition, because the compliant membrane is in contact with the substrate, the interfacial properties have to be studied to avoid the membrane sticking to the substrate after patterning. Using polymeric stamps to manipulate micro- and nano-objects could potentially impact the development of future stencil lithography by precisely controlling the patterning and transfer cycle of stencil masks [124,125]. Such new techniques could prevent the “sticking” problems of compliant stencil masks on the patterning substrate and improve the patterning speed. In addition, the recent development of DNA nanolithography could also improve the patterning scalability and resolution by using DNA origami as stencils. Such low-cost and self-assembled structures have been used as patterning masks for silicon oxide and graphene materials with a feature size of less than 10 nm [126,127].

Another challenge for stencil lithography is the clogging effect. Although stencil lithography can be used to pattern 3D nanostructures with variable patterning height, it changes the feature size of the stencil and could result in the “closure” of the aperture. This is more severe as the feature size decreases. SAM coating has shown the mitigation of the clogging effect. However, it was mainly effective for gold deposition. A cleaning process has been developed to remove the metal deposition on the SiN*x*-based membrane; however, it affects the patterning speed, and requires cleaning before reuse. In addition, corrosive chemicals are required for cleaning, which is not suitable for certain types of stencil mask materials. We believe more research efforts should be made to address such clogging effects in the future.

The precision, orientation, and repeatability of the nanostencil placement are also challenging issues in stencil lithography. The position and displacement of the nanostencil can be precisely controlled with programmable motors and a high-precision stage. The development of piezo actuators allows us to construct a high-precision stage with a resolution of 100 nm [128,129]. Integrating such a high-resolution stage in the stencil lithography system enables the high-precision alignment of nanostencils on the target substrates. Nanometer-scale alignment can also be achieved with the use of multiple lasers and a spectrometer [130]. Combined with polymeric stamps to place/release the stencils [131], it is possible to precisely control the placement, patterning, and release of the nanostencils for next-generation “smart” stencil lithography.

As an ancient method of shadow-mask-based patterning, stencil lithography has proven its capability for nanoscale patterning on various materials and different types of substrates. Stencil lithography masks employ a wide variety of materials, including rigid silicon or SiN*x* masks and flexible membranes such as polyimide, PDMS, or photoresist. Rigid nanostencil masks based on silicon and SiN*_x_* membranes can be fabricated using plasma-enhanced chemical vapor deposition (PECVD) or physical vapor deposition (PVD), and the nanofeatures can be patterned with nanolithography and standard plasma etching techniques. Fabrication processes of such rigid nanostencil masks have been widely studied [132]. However, rigid nanostencil masks with a thickness of hundreds of nanometers could be easily damaged due to their brittle nature. Compliant nanostencil masks including polyimide, PDMS, and photoresist are flexible and more robust. However, the thinnest commercial available polyimide films are ~7.5 µm in thickness, which is very challenging for patterning nanoscale apertures due to the high aspect ratio of thickness/feature size [133]. The fabrication of PDMS nanostencils involves the molding and removal of the top layer. Molding of PDMS with nanofeatures smaller than 500 nm could cause deformation and collapse due to the low elastic modulus [134]. Removing the top layer of molded PDMS is also challenging and always requires additional fabrication steps [79]. Photoresist-based nanostencil masks, on the other hand, can be readily created by spin coating and nanolithography with precise control of the thickness and nanofeature size. The flexibility can also be easily controlled by adjusting the coating thickness or depositing a thin metal layer on top of the photoresist by PVD. Thus, photoresist-based materials are ideal for nanostencil lithography. With these flexible masks, the blurring effect could be effectively reduced. Although there are remaining challenges such as the clogging effect, which could shorten the life span of a stencil lithography mask, efforts have been made by various research groups to develop stencil lithography techniques. More importantly, stencil lithography has unique applications in flexible electronics and bio-devices, with the advantages of scalability, fast, low cost, and freedom from toxic photoresists. Stencil lithography, with its great potential for scalability, is an important addition to the conventional nanofabrication technique.
